# Chemotherapy and Targeted Agents in the Treatment of Elderly Patients with Metastatic Colorectal Cancer

**DOI:** 10.3390/jcm9124015

**Published:** 2020-12-11

**Authors:** Albert Tuca, Rosa Gallego, Ismael Ghanem, Mireia Gil-Raga, Jaime Feliu

**Affiliations:** 1 Department of Medical Oncology, Hospital Clinic, 08036 Barcelona, Spain; atuca@clinic.cat; 2 Department of Medical Oncology, General Hospital of Granollers, 08402 Granollers, Spain; rosagallegosanchez@gmail.com; 3 Department of Medical Oncology, Hospital Universitario La Paz, CIBERONC, 28046 Madrid, Spain; isma_g_c@hotmail.com; 4 Department of Medical Oncology, University General Hospital of Valencia, CIBERONC, 46014 Valencia, Spain; mir_gil@hotmail.com; 5 Cátedra UAM-AMGEN, 28049 Madrid, Spain

**Keywords:** metastatic colorectal cancer, elderly, chemotherapy, targeted therapies, geriatric assessment

## Abstract

Colorectal cancer (CRC) is one of the main causes of cancer death in the elderly. The older patients constitute a heterogeneous group in terms of functional status, comorbidities, and aging-related conditions. Therefore, therapeutic decisions need to be individualized. Additionally, a higher toxicity risk comes from the fact that pharmacokinetics and pharmacodynamics of the drugs as well as the tissue tolerance can be altered with aging. Although the chemotherapy efficacy in metastatic colorectal cancer (mCRC) is similar for older and young patients, more toxicity is presented in the elderly. While the mono-chemotherapy provides the same benefit for young and older patients, doublets front-line chemotherapy improves progression-free survival (PFS) but not overall survival (OS) in the elderly. Furthermore, the benefit of the addition of bevacizumab to chemotherapy in older patients has been shown in several clinical trials, while the clinical data for the benefit of anti-epidermal growth factor antibodies are scarcer. Immunocheckpoint inhibitors could be an appropriate option for patients with microsatellite instability (MSI) tumors. A prior geriatric assessment is required before deciding the type of treatment in order to offer the best therapeutic option.

## 1. Introduction

The incidence and mortality from colorectal cancer (CRC) will increase in the coming decades, mainly due to the progressive aging of the population. The risk of being diagnosed with CRC increases with each decade of life. More than 75% of patients and more than 85% of deaths from CRC will be in patients older than 65 years [1,2]. The overall survival (OS) of patients with metastatic CRC (mCRC) progressively decreases according to the age at which it was diagnosed, from 23–31% three-year survival in patients under 60 to 5–11% in those over 80 years [3].

Therefore, the treatment of mCRC in geriatric patients is a challenge, where the benefit of chemotherapy must be balanced with the higher risk of developing toxicity [4].

Pharmacokinetics and pharmacodynamics of the drugs as well as the tissue tolerance can be altered with aging and secondarily, increase the treatment toxicity [5]. Other factors such as comorbidities and polypharmacy could also increase the risk of toxicity and interactions [6]. Although clinical trials provide most of the data about the efficacy and toxicity of chemotherapy in the elderly, these selected patients are hardly representative of the geriatric population in real life. In addition, there is a lack of data about the impact of chemotherapy in the clinical practice in this population.

Older age itself, without the evaluation of others factors, is often considered as a frailty sign and, therefore, perceived as a risk factor for chemotherapy-induced toxicity [7]. In this scenario, elderly patients could be undertreated, administered suboptimal doses or even omitting treatments, which may decrease efficacy [8,9].

According to the degree of dependence, comorbidities, or geriatric situation, elderly patients constitute a very heterogeneous group. Thus, the election of the most appropriate treatment for every patient should be individualized. Therefore, in these patients, it is important to determine the biological reserve and coping capacity for cancer treatment, so that no patient loses the opportunity to modify the evolution of the disease and, on the other hand, so that no patient is exposed at a disproportionate risk to the real health condition.

## 2. Frailty Definition

Frailty is a complex, multidimensional, and cyclical state of decreased physiological reserve resulting in a reduced capacity for adaptation and adaptability and greater vulnerability to stressors [10,11,12,13,14]. The adverse health outcomes associated with frailty are summarized in disability and functional dependence, cognitive deterioration, increased hospitalization and institutionalization, intolerance to chemotherapy, instability of comorbidities, social exclusion, and decreased survival [10].

The frailty prevalence in persons older than 65 years is between 4% and 17%. Women are almost twice as likely as men to present the condition of frailty (9.2% versus 5.2%). The prevalence of frailty is significantly higher in people older than 80 years [15,16]. Weight loss is frequent in the situation of frailty, either as consequence or as origin of it. However, weight loss should not be considered as a necessary diagnostic criterion because it is also possible to observe frailty in obese people [15].

## 3. Frailty in Oncology

Based on these data, the current consensus of oncologists suggests a mandatory assessment of frailty for all patients over 70 years or those younger people with significant involuntary weight loss in recent months (>5%) [10,15].

The US National Comprehensive Cancer Network and the International Society of Geriatric Oncology recommended adding a geriatric assessment (GA) to standard oncologic evaluation, addressed to detect frailty in elderly patients and help in the decision-making process regarding cancer treatment [17,18]. Most experts propose to evaluate the follow domains of GA in elderly cancer patients: functional status, comorbidity, cognition and mental status, chronic fatigue, social support, and geriatric syndromes. The scientific evidence currently available confirms that GA applied systematically to elderly patients modifies the final decisions of oncologists on the indication or intensity of cancer treatment in 20–45% of cases [19,20,21,22,23,24,25].

Multiple tools have been developed to assess frailty in cancer patients (Vulnerable Elders Survey 13, Flemish version of the Triage Risk Screening Tool, G8 questionnaire, Groningen fragility indicator, Balducci criteria, etc.). Most studies demonstrate the usefulness of geriatric tools and confirm that the most potent individual indicator for the risk of toxicity of cancer chemotherapy is the functional status, evaluated especially by ability to maintain the instrumental activities of daily life, and for overall survival it is the nutritional status [25,26,27].

The geriatric criteria for assessing frailty proposed by Balducci and Exterman [28] classified elderly patients with cancer into three groups. The “fit” group had no significant comorbidities or geriatric syndromes and were independent for activities of daily living (ADL) and instrumental activities of daily living (IADL). The “medium fit” group had fewer than three comorbidities, had no geriatric syndromes, had fewer than four IADI limitations, and no ADL disabilities. The “unfit” group had any of the following conditions: three or more significant comorbidities, geriatric syndromes, more than four limitations of IADL, or ADL disabilities. The recommendations were to treat “fit” patients in the same way as younger patients, adjust treatment doses and/or use monotherapy in “medium fit” patients, and finally, contraindicate cancer treatment and intensify palliative care in “unfit” patients [2,25,28,29,30,31].

In recent years, two tools have been developed specifically aimed at determining the toxicity risk of chemotherapy in elderly patients [32,33]. The Chemotherapy Risk Assessment Scale for High-Age Patients (CRASH) identifies the risk of both hematological toxicity and nonhematological toxicity. This tool includes (a) different components of the GA, such as the comorbidity (Cumulative Illness Rating Scale for Geriatric patient), IADL (Lawton–Brody scale), polypharmacy (number of drugs), nutritional status (Mini Nutritional Assessment), the cognitive situation (Mini Mental State Examination), and the state of mind (Geriatric Depression Scale); (b) biomedical variables (diastolic blood pressure and lactate dehydrogenase); (c) intrinsic potential toxicity of chemotherapy; (d) Eastern Cooperative Oncology Group (ECOG) performance status. The development study of CRASH index identified cut-off points predictive of a high risk of chemotherapy toxicity in cancer patients older than 70 years [18,32]. The Cancer and Aging Research Group (CARG) developed a predictive model of chemotherapy toxicity risk, based on a multicenter cohort of cancer patients older than 65 years, who were systematically administered a GA. The CARG tool finally included different predictive indicators of toxicity risk, such as age, tumor origin, standard or reduced doses of mono or polychemotherapy, hemoglobin, creatinine clearance, falls in the last 6 months, hearing, autonomy to take medications, ability of walking a block, and maintenance of social activity. The combination of the scores assigned to these different variables allowed cut-off points to be established and patients with a potentially unacceptable risk of toxicity to be discriminated [33]. This score was subsequently validated in an external study [34].

## 4. Frailty in Elderly Patients with Metastatic CRC

Different clinical trials in elderly patients with mCRC confirm that the regimens based on 5-Flurouracil (5FU) in mono or polychemotherapy, or biological treatments (panitumumab, bevacizumab, cetuximab) have a similar effect to that observed in younger patients, although they warn about a greater likelihood of adverse effects. These studies included patients with an acceptable biological reserve condition, and no follow-up was established on patients excluded due to their frailty [35].

A recent clinical trial analyzed survival and cancer-specific mortality in elderly patients with early CRC and adjuvant therapy. A GA and the Balducci criteria were used to determine frailty in this study. Of a total of 195 patients, 28% were considered unfit, and the adjuvant therapy was contraindicated, and 29% were considered medium fit, and the adjuvant therapy was adjusted. The five-year survival according to frailty criteria was 74%, 52%, and 27%, for fit, medium fit, and unfit patients, respectively. Most of the unfit patients died early, mainly from non-oncological causes (2). This data provides very interesting information that could be extrapolated to the mCRC.

Different cohort studies confirm a 2.6 times higher risk of mortality in frail patients with metastatic CRC compared with fit patients [31,35]. A multicenter observational cohort study of mCRC patients older than 70 years, treated with chemotherapy or/and bevacizumab during a mean of 5.5 months, recorded severe adverse events in 50% of patients and an overall survival of 8.9 months. In multivariate analysis, poor performance status was predictive of severe toxicity and malnutrition was predictive of a significantly lower survival [27].

The CRASH and CARG tools can be extrapolated and useful for the determination of frailty and the risk of toxicity of chemotherapy in mCRC; although their validation studies did not specifically address for these cases, they included many patients in whom the cancer origin was intestinal (12% and 27% respectively). Recently, a model has been proposed to predict the risk of developing toxicity and early death in chemotherapy-treated colon cancer patients [36]. However, this model has not yet been validated.

A meta-analysis of 37 cohort studies with CRC patients confirms that comorbidity and frailty are strong indicators of survival, and concludes that GA might help the oncologist to make decisions about cancer treatment and the management of geriatric syndromes and multiple comorbidities [35].

On the other hand, a recent study indicates that when the GA is not taken into account for prescribing chemotherapy, 34% of unfit patients are overtreated, which is associated with more grade 3–4 toxicity than those receiving treatment adapted to fragility (42% vs. 31%; *p* < 0.05) (9). In addition, recently, two randomized clinical trials evaluating the impact of GA vs. standard of care on chemo toxicity in older adults with cancer showed the integration of multidisciplinary GA-driven interventions reduced the incidence of grade 3–5 chemo-related toxicity by 10–20% [37,38].

In summary, despite there being few data from research in mCRC, there is a wide consensus that the assessment of frailty should be incorporated into the usual clinical history obtained by the oncologist, in those patients over 70 years of age or in younger people with a weight loss of more than 5% in recent months. The GA is the most complete evaluation tool for assessing frailty. Patients rated as high frailty will have a high risk of toxicity, a lower survival, and most will die from causes other than cancer. In patients with intermediate frailty, there is no formal contraindication to chemotherapy, but dose adjustment and the use of monotherapy should be considered, as well as favoring decision-making shared with the patient based on objective data. Finally, the assessment of frailty can also help to establish a multidisciplinary care plan that includes attention to comorbidity, geriatric syndromes, nutritional status, psychological impact, and socio-family support in those patients with greater needs.

## 5. Chemotherapy for mCRC in the Elderly

Different studies describe older patients receiving both chemotherapy and targeted therapies for mCRC less frequently than young patients [39,40]. However, cytotoxic chemotherapy still remains the mainstay of treatment for patients with mCRC. In fact, several meta-analyses suggest that systemic chemotherapy improves overall survival (OS) compared with best supportive care in patients both young and elderly diagnosed with mCRC [31,41,42,43]. Despite these benefits, it should be noted that both having a gastrointestinal tumor and older age are risk factors for developing grade 3–4 toxicity with chemotherapy [33]. The most frequent treatment-associated toxicity symptoms in the elderly population are diarrhea and neutropenia, although the estimation is heterogeneous because of the diversity in the elderly population [44,45].

The ORR (overall response rate) and OS after 5-FU (5-fluorouracil) treatment are similar for patients aged 70 or older and younger patients. Chemotherapy regimens based on continuous infusion yield better response rates and less toxicities than those based on bolus administration [46,47,48]. Of note, the De Gramont regimen is very well tolerated by elderly patients [44].

Several studies have shown that capecitabine alone is at least as effective as bolus 5-FU in elderly patients with good overall health status [45,46,47,48]. The optimal capecitabine dosage for patients older than 70 is unknown, especially for women, because the age-associated reduction in creatinine clearance can worsen toxicity [48].

While the benefit of fluoropyrimidines in response rate and overall survival is similar for older and younger patients [46,47,48,49], the benefit of oxaliplatin- or irinotecan-based combinations is under discussion [49,50]. In fact, neither of the two phase III clinical trials, conducted in the elderly, comparing fluoropyrimidines versus their combination with oxaliplatin (FOCUS 2) [51] or irinotecan (FFCD2001-02) [52] showed an increase in OS or progression-free survival (PFS) (Table 1). However, in both studies, the response rate (RR) achieved with doublets of chemotherapy was superior to that achieved with monotherapy. Moreover, a subsequent meta-analysis based on these trials and on the data of an elderly subgroup of trials without limit of age suggested that doublets in the elderly prolonged the PFS (Hazard Rate (HR) = 0.82; CI: 0.72–0.93), but not OS (HR = 1.00; 95% CI: 0.89–1.13) [53].

Therefore, when the goal of the treatment is to maintain or improve the quality of life and increase the survival while minimizing the chemotherapy toxicity, monotherapy would be the best option of treatment. Conversely, for fit patients who aim to obtain maximum tumor response to control symptoms or achieve resectability for metastatic or advanced disease, doublet chemotherapy seems to be the treatment of choice. In any case, the different therapeutic options should be discussed with the patient so that they can participate in the decision.

Trifluridine/tipiracil (TAS-102) is an oral monotherapy indicated for treatment of pretreated mCRC. In the pivotal phase III study, TAS-102 showed a significant improvement in OS (from 5.3 to 7.1 months) and PFS (from 1.7 to 2.0 months), with a favorable safety profile in heavily pretreated patients. The main toxicity was myelosuppression [54]. A posterior subgroups analysis highlighted the benefits of TAS-102 in elderly patients [55]. In addition, the results of the open-label expanded-access program in USA confirmed no differences in safety profile for elderly versus younger patients [56].

## 6. Targeted Therapies in mCRC

As in other metastatic colorectal aspects, the elderly population is also underrepresented in clinical trials that analyze targeted therapies. However, we have some data in the literature about the use of targeted therapies in this population that can help us to make decisions in the daily practice.

### 6.1. Antiangiogenic Therapy

#### 6.1.1. Bevacizumab

The randomized phase III clinical trial AVEX examined the efficacy and safety of treatment with capecitabine with or without bevacizumab (bvz) specifically in population ≥ 70 years in first-line treatment of mCRC. The combination showed a significant benefit in the PFS (primary objective of the study) with 9.1 vs. 5.1 months (*p* < 0.0001); no differences in OS were observed (Table 2). Adverse effects (AEs) were more frequent with the combination [57]. A randomized phase II clinical trial conducted mostly in mCRC patients aged 65 years and older showed a significant benefit for the addition of bevacizumab to 5-FU/LV (leucovorin) in terms of PFS (9.2 vs. 5.5 months; HR = 0.50; *p* = 0.0002) and a trend to improve the OS (16.6 vs. 12.9 months; HR = 0.79; *p* = 0.16) [58] (Table 2). The PRODIGE-20 is another phase II clinical trial that randomized mCRC patients 75 or older to chemotherapy-alone or chemotherapy–bvz. The primary endpoint was composite, based on efficacy (tumor control, stable disease, or objective tumor response and the absence of a decrease in the Spitzer Quality of life (QoL) index) and safety (absence of severe cardiovascular toxicities and unexpected hospitalization). The efficacy and safety criteria were met in 58% and 71% of the patients, respectively, in the chemotherapy-alone arm and in 50% and 61% for the chemotherapy plus bvz arm. Median PFS was 7.8 and 9.7 months for the chemotherapy-alone and the bvz–chemotherapy arms, respectively [59] (Table 2).

Some subgroup analyzes from various randomized clinical trials has been performed in order to explore the benefit from bvz in the elderly. In the first-line setting, the MAX trial subanalysis, with three treatment arms (capecitabine vs. capecitabine plus bvz vs. capecitabine plus bvz plus mitomycin C), showed a benefit in PFS for bvz (8.8 vs. 5.8 months) in 99 patients of 75 years or more. No differences in toxicity were observed according to age [60]. Moreover, Fyfe and collaborators carried out the analysis of the subgroup ≥ 65 years on the phase III clinical trial AVF2107. The median OS was 14.9 months for the chemotherapy with irinotecan–5-FU–LV (IFL) alone arm versus 24.4 months for the bvz combination arm. The incidence of adverse events did not increase in older patients treated with the antiangiogenic drug [61]. Although in these studies the tolerance was favorable, it should be noted that the administration of bvz in older patients has been associated in some trials to a higher risk of arterial thrombotic phenomena. The pooled analysis of four randomized clinical trials comparing chemotherapy with chemotherapy–bvz performed by Cassidy and colleagues found a benefit in PFS in all age subgroups [62]. The incidence of arterial thromboembolic adverse events was higher in patients aged ≥65 years and ≥70 years. A similar analysis of seven randomized trials showed a similar benefit in PFS and OS in all age subgroups [63].

In the observational study BRITE (Bevacizumab Regimens Investigation of Treatment Effects), which included 1953 patients treated with chemotherapy plus bvz in the first-line setting, 896 patients were 65 years or older and 363 were 75 years or older. The median of PFS was similar among all age subgroups, but the OS decreased with age. Arterial thromboembolic events were the only adverse event that increased with age [64]. In addition, this risk was even higher in patients with a history of arterial thrombotic events [65]. The other large observational study BEAT (Bevacizumab Expanded Access Trial), with a similar design to the previous one, included 499 patients between 65 and 74 years and 129 aged 75 years or more. The results showed a decrease in the use of combinations of chemotherapy as age increased. However, there was no increase in the incidence of AEs with age [66].

#### 6.1.2. Aflibercept

In the VELOUR trial [67] that randomized 611 patients to receive FOLFIRI (5-FU–LV–irinotecan) with or without aflibercept in the second-line treatment of chemotherapy after progression to a regimen with oxaliplatin, 34% of the patients in the combination arm were 65 years or older and only 5% were 75 years or more. In the post hoc analysis according to age, there were no significant differences in OS among patients < 65 years versus ≥ 65 years, however the incidence of adverse effects was higher in the older group (mainly diarrhea, dehydration, asthenia, and weight loss) [68].

#### 6.1.3. Regorafenib

Regorafenib, a multi-kinase inhibitor, has showed, in the phase III CORRECT trial, an improvement in PFS and OS compared with placebo in previously heavily treated mCRC patients. However, few data are disposable on efficacy and toxicity in the elderly. A post hoc analysis of the CORRECT trial looking at age subgroups (<65 years vs. ≥65 years) showed no significant differences in efficacy or in toxicity. In this study, the population ≥ 75 years was underrepresented (8% of the total of patients included) [69]. In the CONSIGN trial (Phase IIIb, single-arm treatment with regorafenib in patients with mCRC after progression to standard therapy) with 2872 patients included, no differences were observed in the efficacy and toxicity profile according to age (<65 years vs. ≥65 years) [70].

A phase II multicenter study evaluated regorafenib in mCRC patients over 70 years previously treated. PFS and OS were 2.2 and 7.5 months. Grade 3–4 treatment-related adverse events were observed in 83% of the patients, and asthenia was the most frequent one. A trend to discontinue the study due to toxicity was observed among those older than 80, ECOG ≥ 1, and with impaired baseline autonomy [71].

In another single-arm phase II study that included 47 elderly patients (median age of 80 years), who were not candidates to receive intensive chemotherapy, the efficacy and safety of regorafenib as the first line of treatment was analyzed. The PFS and OS medians were 5 and 16.7 months, respectively, and 25% of the patients interrupted the treatment due to toxicity. Two patients presented grade 5 toxicity, and the incidence of hypertension and asthenia 3–4 grade was 37.5% and 35%, respectively [72].

#### 6.1.4. Ramucirumab

In the phase III RAISE study (FOLFIRI with or without ramucirumab after progression to the first line of chemotherapy with bvz), 209 patients aged 65 or older were included, of whom 51 were at least 75 years old. In the subgroup analysis (<65 years vs. ≥65 years), the OS benefit and the toxicity profile with ramucirumab were similar in both groups [73].

### 6.2. Anti-EGFR Therapy

The experience with anti epidermal growth factor receptor (anti-EGFR) therapies is limited to phase II clinical trials and phase III subanalyses that were not designed specifically for the elderly.

#### 6.2.1. Cetuximab

In a combined analysis of the efficacy and safety according to the age of the patients included in the OPUS and CRYSTAL trials in the first line of treatment, a significant survival benefit was observed for the combination of cetuximab with FOLFOX (5-FU–LV–oxaliplatin) or FOLFIRI vs. chemotherapy alone in patients older than 70 years (PFS of 8.9 vs. 7.2 months and OS of 23.3 vs. 15.1 months). The toxicity was greater in the combination arm without statistically significant differences (mainly diarrhea and cutaneous toxicity) [74]. On the other hand, in the observational trial ERBITAG (that evaluated the efficacy and safety of cetuximab in combination with chemotherapy in the first line of treatment in KRAS wild-type (wt) patients), ORR and PFS of the population ≥ 75 years without comorbidity were similar to those of the young population. However, in those patients with one or more comorbidity, time to treatment failure was lower [75].

Several phase II studies have been conducted with cetuximab specifically in the elderly population. It is worth highlighting a study with 66 patients aged 70 years or more that evaluated the efficacy and safety of treatment with cetuximab and capecitabine. In 27 of the patients included, the dose of capecitabine was reduced to 1000 mg/m, two every 12 h. After dose reduction, the scheme treatment was well tolerated and the main toxicities were rash and paronychia (28.2% and 7.7% grade 3, respectively). The ORR was 48.3%, and the median of PFS was 8.4 months [76]. This treatment scheme was compared with cetuximab monotherapy in a population ≥ 75 years or ≥70 years with functional dependence in KRAS/ BRAF wt patients. Although only 24 patients were included, results showed an ORR benefit in the combination arm (31% vs. 9%) with acceptable toxicity, being skin toxicity, diarrhea, and infections the most frequent [77]. Cetuximab monotherapy has also shown some efficacy in monotherapy in population ≥ 74 years, KRAS wt (ORR 51.6%) with an acceptable tolerance in a single-arm study that included 31 patients [78].

#### 6.2.2. Panitumumab

PANDA study is a randomized phase II trial comparing FOLFOX–panitumumab vs. 5-FU–LV–panitumumab in untreated RAS-BRAF wild-type patients aged ≥70 years. Median PFS was similar in both arms (9.6 and 9.1 months, respectively), although grade 3–4 toxicity was more frequent in the FOLFOX–panitumumab arm, particularly diarrhea and neutropenia [79]. Analysis of subgroups of elderly patients treated in a trial that compared panitumumab with best supportive care revealed no significant difference for toxicity or efficacy compared with younger patients [80]. Similarly, a post hoc analysis according to the age of the randomized clinical trial PRIME that compared in first-line FOLFOX4 plus panitumumab and FOLFOX4 [81] was performed. In the RAS wild-type population, 189 patients were 65 years of age or older (of which 34 were older than 75 years). Both the median PFS (primary end point) and median OS in the population over 65 years were superior in the combination arm without significant statistically differences (PFS 9.7 vs. 9.2 months, HR = 0.89, *p* = 0.43, and OS 26.6 vs. 17.4 months, HR = 0.80, *p* = 0.15). In the population > 75 years of age, the median OS was higher in the FOLFOX4 arm without significant differences (OS 11.1 vs. 15.9 months, HR = 1.14, *p* = 0.71). The incidence of serious adverse effects related to treatment increased with age, especially in the FOLFOX–panitumumab arm. These results are consistent with a preplanned post hoc analysis according to age (<65 vs. ≥65 years) from the second-line clinical trial 20050181, comparing FOLFIRI–panitumumab vs. FOLFIRI. In this study, no differences for PFS or OS were found regarding the age. A higher incidence of grade 3/4 AE was observed in the panitumumab–FOLFIRI arm in a similar way in the two age groups [80].

The phase II clinical trial FRAIL evaluated specifically the efficacy of panitumumab monotherapy in a first-line setting in population ≥ 70 years with KRAS exon 2 wt. In the analysis of the RAS wild-type population, the medians of PFS and OS were 7.9 and 12.3 months, respectively. The treatment was well tolerated, with 30% grade 3 toxicity related to panitumumab [81].

Considering these data as a whole, these results show that age alone is not a criterion for not administering a targeted therapy, although its use in the elderly population should be carefully monitored.

### 6.3. Immune Checkpoint Inhibitors

Data published in recent years show the efficacy of immune checkpoint inhibitors (ICI) in patients with mCRC who present a deficiency in the DNA repair system (Mismatch repair deficiency/high level microsatellite instability (MMRd/MSI-H)), a subtype that accounts for approximately 5% of the patients.

The trials that analyzed the efficacy of ICI in this subpopulation included a considerable percentage of patients ≥ 65 years, although the efficacy and safety data in this subgroup are still scarce. With pembrolizumab (Anti-PD-1) in monotherapy in patients with mCRC and MSI-H, two studies stand out. The first is the phase II Keynote 164 study [82] that evaluated the efficacy and safety of the drug in 124 patients in two cohorts (cohort A ≥ 2 previous lines and cohort B ≥1 previous line). In the study, 28% of patients were aged >65 years in cohort A and 38% in cohort B. The RR (primary endpoint) was 33% in both cohorts. With a median follow-up of 31.4 months, the PFS in cohort A was 2.3 months and the OS was 31.4 months. In cohort B, with a 24 months follow-up, the PFS was 4.1 months and the median OS was not reached. A good tolerance was observed with 13% and 16% of adverse events grade (AEG) ≥ 3 in each cohort. The second study Keynote-177 [83] was presented by Andre T. at the de ASCO 2020 conference. It is a phase III study with 307 patients that compared pembrolizumab vs. chemotherapy +/− bevacizumab or cetuximab (investigator’s choice) in the first line of treatment. Of the 307 patients included, 29% were older than 70 years. The results showed a clear benefit of pembrolizumab in PFS (one of the primary objectives of the study) with a PFS rate at 12 months of 55% vs. 37% (HR 0.60; *p* = 0.0002). In this case, the analysis by subgroups included age and observed a significant benefit with pembrolizumab in patients aged ≤70 years (HR 0.52 (0.37–0.75)) and a favorable trend to pembrolizumab in those aged >70 years without reaching statistical significance (HR 0.77 (0.46–1.27)). Regarding the toxicity reported, the percentage of AE G ≥ 3 with pembrolizumab decreased 44% with respect to chemotherapy. Data published on the ESMO 2020 conference showed a significantly increase in the quality of life with pembrolizumab in the overall study population compared with chemotherapy.

Nivolumab (Anti-PD-1), both in monotherapy and in combination with ipilimumab (anti-CTLA-4), has also shown efficacy in pre-treated patients with mCCR and MSI-H. In both studies, more than 30% of the patients included were ≥65 years of age. The Checkmate 142 [84] study analyzed the efficacy and safety of nivolumab in monotherapy in 74 heavily pretreated patients, observing an RR of 32% and a PFS and OS rate at 12 months of 50.4% and 73.4%, respectively. The percentage of treatment-related adverse effects (TRAEs) G ≥ 3 was 20.3%. The study that evaluated the combination with nivolumab and ipilimumab in 119 patients published by Overman [85] showed an RR of 55% and a PFS and OS rate at 12 months of 71% and 85%, respectively, at expense of greater toxicity (TRAEs G ≥ 3 of 32%).

In CRC, we still do not have ICI studies focused exclusively on the elderly population, however, the data published on other solid tumors may be useful. Nosaki et al. [86] published an exploratory pooled analysis of the efficacy and safety of pembrolizumab monotherapy versus chemotherapy in elderly patients (aged ≥75 years) with non-small cell lung cancer (NSCLC) from the Phase 2/3 KEYNOTE-010 study, Phase 3 KEYNOTE-024 study, and Phase 3 KEYNOTE-042 study. The 12 months OS was superior with pembrolizumab vs. chemotherapy in elderly patients with PD-L1 Tumor Proportion Score (TPS) ≥ 1% (HR: 0.76 (95% CI: 0.56–1.02)) and with PD-L1 TPS ≥ 50% (HR: 0.40 (95% CI, 0.25–0.64)). TRAEs were 68% with pembrolizumab versus 94% with chemotherapy, and immune-mediated adverse events (IMAEs) and infusion reactions (all grades) were 25% with pembrolizumab versus 7% with chemotherapy.

Some studies include a geriatric assessment. Welaya et al. [87] evaluated the associations of GA domains with treatment-related outcomes in 28 older adults with solid tumors receiving ICIs. Seventy-five percent had at least one GA domain impairment, and patients with any instrumental activities of daily living (IADL) impairment received fewer cycles of ICI (median: 2.0 vs. 7.0 cycles, *p* = 0.02). Finally, Sakakida et al. [88] evaluated retrospectively safety and tolerability of ICI in elderly and frail patients with advanced malignancies. A total of 58 patients were aged ≥75 years. No significant difference was found in the development of IMAEs, hospitalization, and treatment discontinuation due to IMAEs between elderly and young populations, nevertheless, more critical complications and fatal IMAEs were observed among elderly patients with high frailty.

## 7. ESMO Magnitude of Clinical Benefit

The ESMO/MCBS 1.1. scale asses the magnitude of clinical benefit for anticancer treatments. The score obtained when this scale was applied to the two phase III trials, which compared the addition of irinotecan or oxaliplatin, respectively, to fluoropyrimidines, was 1, the lowest score because neither study achieved the primary objective (a PFS increase) [51,52] (Table 1). In addition, doublets were associated to a higher toxicity rate.

On the other hand, the ESMO/MCBS 1.1. scale could be applied only in two of the three randomized clinical trials exploring the benefit of the addition of bvz to chemotherapy in the elderly [57,58] (Table 2). In both cases, the score obtained was 3. It should be noted that the primary objective was benefit in PFS for the phase III [58] and OS for the phase II [58]. There was no downgrade because of a higher toxicity or upgrade due to an improvement in quality of life. In the third trial [60], the main variable, assessed 4 months after randomization, was composed of tumor control (radiological response or stabilization) without decrease of quality of life in absence of severe cardiovascular toxicities or an unexpected hospitalization. This type of composite variable provides interesting information about the balance between efficacy and quality of life/toxicity in the elderly, although the ESMO/MCBS 1.1. scale cannot be applied. However, the scale can be applied when the main objective of the clinical trial is to demonstrate an increase in quality of life or a decrease in toxicity.

The ESMO/MBCS 1.1. scale could not be applied to the anti-EGFR phase II clinical trials, because of the early closure secondary to lack of recruitment in the SAKK 41/10 trial [77], and because a formal comparison between the two arms was not planned in the PANDA study [79].

## 8. Global Interpretation

From the methodological point of view, it is worth highlighting the definition of elderly is widely variable, ranging from 65 to 75 years old among the different clinical trials. Moreover, the fragility criteria were rarely established according to a rigorous GA, resulting in a heterogenous population that could condition the clinical trial results. Additionally, the initial chemotherapy doses were arbitrarily decreased in some clinical trials [51,52], which potentially could have an impact on the efficacy and survival: a dose adjustment in a vulnerable patient can be adequate, but the same adjustment in a fit patient could imply an undertreatment. Other aspect to take into account in the clinical trials design is that the primary objectives are often overly conformist, limited to demonstrating an increase in PFS [51,52,57,59,79]. However, this benefit probably has no value without a parallel significant OS increase and/or quality of life improvement. In addition, since most of the older patients prioritize quality of life over quantity of life, effort must be made to adapt the objectives of clinical trials by focusing not only on OS but also on variables such as quality of life and functional status. On the other hand, there is a potential selection bias in clinical trials in the elderly, where the patients usually have no comorbidities with a significantly better health condition than the global geriatric population. Furthermore, the close follow-up carried out in clinical trials allows the toxicities to be detected earlier with less severe consequences than in the clinical practice [89]. In addition, the pharmacokinetics and pharmacodynamics of the drugs as well as the tissue tolerance can be altered with aging. Thus, the clinical trials do not allow a reliable extrapolation of clinical data from clinical trials to real-life patients in the elderly and, consequently, there is a need to perform specific trials in this population.

Deciding the appropriate treatment is one of the most important actions in oncology, which can become a main challenge in the older mCRC patient. GA can be a very useful tool to achieve this objective. Moreover, there are other tools that can predict the risk of developing severe chemotherapy toxicity [32,33,36,90,91]. Conducting the GA is very useful not only before starting the treatment but also during or at the end of the chemotherapy in order to evaluate the impact of the treatment on health. This allows the diagnosis of less severe toxicities, sequelae, or even helps to determine a functional deterioration and a decrease in quality of life secondary to the treatment.

## 9. Conclusions

The chemotherapy efficacy in the elderly is similar to that of young patients, however more toxicity could be presented in the older patient. Therefore, it is critical to identify those patients with higher risk of developing serious toxicity through a geriatric assessment that includes at least functional status, comorbidity, nutritional status, life expectancy, and chemotherapy toxicity risk. For unfit elderly ECOG PS 3–4 patients, palliative care without chemotherapy administration would be the best option. For fit patients, with good functional status and without significant comorbidity, the same treatment as in younger patients (FOLFOX or FOLFIRI) could be administrated, particularly when the goal is to obtain maximum tumor response to control symptoms or achieve resectability for metastatic or advanced disease. For unfit but not frail patients, a monotherapy treatment, such as 5-FU–LV or capecitabine, or a doublet with dose reduction should be discussed with the patient after a careful evaluation with the previously mentioned tools. On the other side, the association of target therapies such as bevacizumab or anti-EGFR to chemotherapy could be considered after a risk–benefit assessment. Immunocheckpoint inhibitors could be an appropriate option for patients with microsatellite instability (MSI) tumors. Finally, the promotion of high-quality clinical trials evaluating OS but also incorporating important objectives of older adults with cancer such, as quality of life or functional status, is warranted.

## Figures and Tables

**Table 1:** Phase III trials comparing fluoropyrimidines monotherapy or doublet chemotherapy in metastatic colorectal cancer in elderly patients.

Author (Reference)	No. Pts	Treatments	HR PFS/OS < 0.8	Adequate Control Arm	Any Change in Primary Endpoint or Sample Size	Achieved Pre-Specified Objective	Quality Design	ESMO/MCBS (PFS)
Seymour MT [52]	459	5-FU, Cape FUOX, CAPOX	0.84/0.99	Yes	No	No	2	1
Aparicio T [53]	282	5-FU–LV FOLFIRI	0.84/0.96	Yes	No	No	2	1

Abbreviations: Pts, patients; HR, Hazard rate; PFS, progression-free survival; OS, overall survival; ESMO, (European Society for Medical Oncology); MCBS, (Magnitude of Clinical Benefit Scale); 5-FU, 5-fluorouracil; Cape, capecitabine; FUOX, 5-fluorouracil–oxaliplatine; LV, leucovorin; FOLFIRI, 5-fluorouracil–leucovorin–irinotecan.

**Table 2:** Randomized phase II–III trials with bevacizumab in metastatic colorectal cancer in elderly patients.

Author (Reference)	No. Pts	Treatments	HR PFS/OS < 0.8	Adequate Control Arm	Any Change in Primary Endpoint or Sample Size	Achieved Pre-Specified Objective	Quality Design	ESMO/MCBS (PFS)
Cunningham D [58]	280	Cape Cape–Bvz	0.53/0.79	Yes	No	Yes	3	3
Kabbinavar FF [59]	209	5-FU–LV 5-FU–LV–Bvz	0.50/0.79	Yes	No	Yes	3	3
Aparicio T [60]	102	Any QT Any QT–Bvz	0.79/0.73	Yes	No	Yes	3	*

Abbreviations: Pts, patients; HR, Hazard rate; PFS, progression-free survival; OS, overall survival; ESMO, (European Society for Medical Oncology); MCBS, (Magnitude of Clinical Benefit Scale); Cape, capecitabine; Bvz, bevaciumab; 5-FU, 5-fluorouracil; LV, leucovorin; FOLFIRI, 5-fluorouracil–leucovorin–irinotecan. * ESMO/MCBS for OS not evaluable because the primary objective in both trials was PFS.
